# Histone Demethylase MoRph1 Regulates Fungal Development, Pathogenicity, and DNA Damage Repair in *Magnaporthe oryzae*

**DOI:** 10.3390/jof12050338

**Published:** 2026-05-05

**Authors:** Dong Li, Chun Yin, Wanying Zhao, Youyang Wang, Shoujian Zang, Wenzhi Wang, Youxiong Que, Qibin Wu, Weixiang Wang

**Affiliations:** 1 State Key Laboratory of Tropical Crop Breeding, Institute of Tropical Bioscience and Biotechnology, Chinese Academy of Tropical Agricultural Sciences, Sanya 572024, China; lidong09062023@163.com (D.L.); zhaowanying@catasitbb.cn (W.Z.); zangshoujian2020@163.com (S.Z.); wangwenzhi@catasitbb.cn (W.W.); queyouxiong@126.com (Y.Q.); 2 Beijing Key Laboratory of New Technology in Agricultural Application, National Demonstration Center for Experimental Plant Production Education, Beijing University of Agriculture, Beijing 102206, China; wangyouyang@bua.edu.cn; 3 Vocational and Technical College, Inner Mongolia Agricultural University, Baotou 014109, China; yinchun20071111@163.com

**Keywords:** *Magnaporthe oryzae*, appressorium, DNA damage response, epigenetic regulation, H3K36me3, chromatin remodeling, virulence

## Abstract

Histone demethylases regulate epigenetic modifications and DNA damage repair in fungal pathogens, yet their specific functions in *Magnaporthe oryzae* remain poorly understood. This study identifies MoRph1, a JmjC domain-containing histone demethylase that interacts with the COMPASS complex. Targeted deletion of *MoRph1* resulted in significantly reduced vegetative growth, impaired conidiation, and defective appressorium formation. The mutant displayed compromised appressorial turgor pressure due to delayed degradation of glycogen and lipid reserves, leading to inefficient host penetration and attenuated virulence on rice and barley. MoRph1 localized to the nucleus, and its absence caused increased nuclear abnormalities under DNA damage stress, suggesting impaired genome stability maintenance. Biochemical analysis confirmed that MoRph1 specifically demethylates histone H3 lysine 36 trimethylation. Transcriptome analysis revealed altered expression of genes associated with DNA replication, mismatch repair, and oxidative stress response. These results establish MoRph1 as a crucial epigenetic regulator coordinating fungal development, infection structure function, energy mobilization, and DNA damage repair. This study underscores the importance of chromatin-level regulation in fungal pathogenicity and provides a foundation for future evaluation of MoRph1 as a potential antifungal target.

## 1. Introduction

Rice (*Oryza sativa*) serves as a staple food crop for over half of the global population, particularly in Asia. However, rice production faces severe threats from rice blast disease, caused by the ascomycete fungus *Magnaporthe oryzae* [1]. This devastating pathogen initiates infection through the formation of specialized dome-shaped infection structures called appressoria on the host surface, which generate substantial turgor pressure to physically breach the plant cuticle and cell wall [2,3,4]. Subsequent colonization of host tissues results in characteristic necrotic lesions and significant economic losses [5,6]. Elucidating the molecular mechanisms underlying *M. oryzae* pathogenicity is therefore essential to developing sustainable disease management strategies. Previous studies have identified numerous genetic determinants regulating morphological transitions, including conidiation and appressorium development [7,8]. Key regulators such as COM1, which is required for conidial morphology and full virulence [9], and CHS6, a chitin synthase gene controlling infection-specific morphogenesis [10], underscore the critical importance of structural development in disease progression.

Recently, attention has shifted toward chromatin-mediated regulation of fungal virulence. Histone modifications, including methylation and acetylation, play pivotal roles in orchestrating gene expression during infection. In *M. oryzae*, MoSnf5, a subunit of the SWI/SNF chromatin remodeling complex, controls conidiation and appressorium function by altering chromatin accessibility [11]. Additionally, JmjC domain-containing histone demethylases MoJmj1 and MoKMT2H modulate developmental transitions and infection processes, likely through demethylation of H3K4me3 or H3K36me3 residues [12,13,14]. These enzymes exhibit functional analogy to Rph1 in *Saccharomyces cerevisiae*, a damage-responsive H3K36 demethylase that maintains genome stability [15]. Rph1 interacts with the DDR kinase Rad53 and undergoes phosphorylation under genotoxic stress, thereby derepressing DNA repair genes such as PHR1 [16,17,18], illustrating the intimate link between histone demethylation and stress adaptation mechanisms.

The DNA damage response (DDR) is essential for maintaining genome integrity, particularly during pathogen–host interactions when fungi encounter host-derived reactive oxygen species and DNA-damaging agents [19,20]. Although DDR pathways have been extensively characterized in mammalian systems [21,22,23], their roles in plant-pathogenic fungi are only beginning to emerge. Nuclear dynamics observed during appressorium formation suggest active chromatin remodeling and nuclear envelope reorganization are required for successful infection [24]. Beyond chromatin regulation, post-transcriptional mechanisms contribute significantly to fungal adaptation. The RNA-binding protein MoGrp1 regulates splice-site selection and virulence-related transcript variants [25], while SR protein homologs influence infection-specific isoform production [26]. Transcriptome analyses further reveal enrichment of long non-coding RNAs and alternative splicing events during infection stages [27]. Post-translational modifications provide an additional layer of regulatory complexity. For instance, disruption of α-1,3-mannosyltransferase ALG3 impairs N-glycosylation of secreted effectors and compromises pathogenicity [28]. The MoB56/PP2A phosphatase complex regulates infection-related signal transduction, and genome-wide effector prediction continues to uncover novel virulence determinants in *M. oryzae* [28].

Collectively, these findings demonstrate that *M. oryzae* virulence depends on a sophisticated, multilayered regulatory network spanning chromatin remodeling, DNA damage repair, RNA processing, and protein modification. However, the specific functions of histone demethylases in integrating chromatin-level control with DNA damage repair and infection processes remain poorly understood in this important pathogen.

*MoRph1* (*MGG_09186*) is predicted to encode a JmjC domain-containing histone demethylase, but its biological function in phytopathogenic fungi has not yet been characterized. Notably, MoRph1 shows similarity to Saccharomyces cerevisiae Rph1, an H3K36 demethylase implicated in genome stability and DNA damage response [15,16,17,18]. Based on this, we investigated the role of MoRph1 in fungal development, pathogenicity, and stress adaptation in *M. oryzae*.

## 2. Materials and Methods

### 2.1. Fungal Strains and Culture Conditions

The *M. oryzae* wild-type strain P131 [29] and derived transformants (ΔMoRph1-1, ΔMoRph1-2, MoRph1C, and MoRph1-GFP reporter strain) were maintained on oatmeal tomato agar at 28 °C for routine culture. For conidiation induction, strains were grown on OTA plates at 28 °C for 7–10 days. Conidia were harvested by flooding cultures with sterile distilled water and gentle agitation with a sterile spreader, followed by filtration through two layers of Miracloth (Calbiochem, San Diego, CA, USA) to remove mycelial debris. Conidial concentrations were determined using a hemocytometer (Neubauer, Germany) and adjusted to the required density (1 × 10^5^ conidia/mL for most assays) with sterile water.

For mycelial biomass production, 1 × 10^6^ conidia/mL were inoculated into 100 mL liquid CM in 250 mL Erlenmeyer flasks and incubated at 28 °C with shaking at 160 rpm for 48 h. Mycelia were collected by vacuum filtration using Whatman No. 1 filter paper, immediately frozen in liquid nitrogen, and stored at −80 °C for genomic DNA (gDNA), and total RNA [11,30]. For transformant selection, hygromycin B (Calbiochem) was added to solid media at a final concentration of 250 μg/mL, and geneticin (G418 sulfate, Sigma-Aldrich, St. Louis, MO, USA) at 400 μg/mL. All chemicals were of analytical grade or higher purity unless otherwise stated.

### 2.2. Identification and Bioinformatic Analysis of MoRph1

*MoRph1* (*MGG_09186*) was identified from the *Magnaporthe oryzae* P131 genome based on homology to the *Saccharomyces cerevisiae* histone demethylase Rph1. The amino acid sequence of yeast Rph1 was used as a query for BLASTP searches against the *M. oryzae* genome database. Conserved domains in MoRph1 were predicted using InterProScan. Domain organization was visualized based on the InterPro annotation.

### 2.3. Construction of Gene Deletion, Complementation, and Fluorescent Reporter Strains

The *MoRph1* (*MGG_09186*) gene deletion mutant (Δ*MoRph1*) was generated via homologous recombination as previously described [31] with minor modifications. The resultant deletion construct was transformed into protoplasts of wild-type strain P131 using polyethylene glycol (PEG)-mediated transformation [31]. Putative transformants were initially selected on CM plates containing hygromycin B (250 μg/mL) and subsequently screened by PCR using gene-specific primers flanking the target locus to confirm homologous integration and complete gene replacement. Two independent deletion mutants (ΔMoRph1-1 and ΔMoRph1-2) were obtained and used for subsequent experiments to eliminate strain-specific artifacts.

For subcellular localization analysis, the full-length MoRph1 coding sequence (excluding the stop codon) was amplified from P131 cDNA using specific primers. The amplified fragment was digested with BamHI/KpnI and inserted into the corresponding sites of the p-rGTN vector [32] which drives expression under the constitutive RP27 promoter and encodes a C-terminal green fluorescent protein (GFP) fusion. The resulting MoRph1-GFP construct was verified by Sanger sequencing (Tsingke, Beijing, China) and transformed into P131 protoplasts. Transformants were selected on CM plates containing 400 μg/mL geneticin and screened for GFP fluorescence using confocal microscopy.

To generate the complemented strain (MoRph1C), the full-length MoRph1 ORF (including its native promoter, 1.5 kb upstream of the start codon) was amplified and cloned into the YIP102 vector (containing 3 × Flag and HA epitope tags and G418 resistance gene). The construct was transformed into ΔMoRph1-1 protoplasts, and complementation was verified by phenotypic restoration (growth rate, conidiation, virulence).

### 2.4. Phenotypic and Growth Assays

Colony morphology and growth rate were assessed by spotting 5 μL of conidial suspension (1 × 10^5^ conidia/mL) at the center of OTA or CM agar plates (90 mm diameter). Plates were incubated at 28 °C for 7 days, and colony diameter was measured in two perpendicular directions daily using a digital caliper (Mitutoyo Corporation, Kawasaki, Japan). Each strain was tested in triplicate, and experiments were repeated independently at least three times.

Conidiation was quantified by harvesting conidia from 10-day-old OTA cultures as described above. The conidial suspension was concentrated by centrifugation at 8000× *g* for 5 min, resuspended in 1 mL sterile water, and counted using a hemocytometer under a light microscope (Nikon Ni-U, Nikon, Tokyo, Japan) at ×400 magnification.

Conidial germination and appressorium formation assays were performed on hydrophobic glass coverslips. Freshly harvested conidia were resuspended in sterile distilled water at 1 × 10^5^ conidia/mL, and 50 μL droplets were placed on coverslips placed in moist chambers to maintain 95% relative humidity. The chambers were incubated at 25 °C in the dark. Germination and appressorium formation were monitored at 4, 8, and 12 h post induction (hpi) by mounting coverslips on glass slides and observing under a Nikon Ni-U microscope [33]. At least 100 conidia were counted per replicate, with three replicates per strain.

### 2.5. Appressorium Turgor Pressure Measurement

Appressorial turgor was assessed by cytorrhysis assays using hyperosmotic glycerol solutions [34]. Conidial suspensions (1 × 10^5^/mL) were incubated on hydrophobic coverslips for 24 h at 25 °C to allow appressorium maturation. Glycerol solutions (0.5 M–2 M) were prepared in distilled water, and 50 μL aliquots were applied directly to the coverslips. After incubation for 10 min at room temperature, the number of collapsed appressoria (cell wall invagination or loss of structural integrity) was enumerated under bright-field microscopy (Nikon Ni-U). At least 100 appressoria were examined per treatment, with three independent replicates for each strain and concentration. Turgor pressure was indirectly reflected by the percentage of collapsed appressoria at each glycerol concentration.

### 2.6. Histochemical Staining of Energy Reserves

Glycogen and lipid body dynamics were monitored during appressorium development at 0, 4, 8, and 12 hpi. For glycogen detection, coverslips with conidia/appressoria were stained with an iodine solution (2% KI/I_2_, *w*/*v*; Sigma-Aldrich) for 5 min at room temperature, rinsed briefly with distilled water, and observed immediately under bright-field microscopy (Nikon Ni-U). Glycogen appears as dark brown deposits.

For neutral lipid visualization, samples were stained with 10 μg/mL Nile Red (Sigma-Aldrich; stock solution 1 mg/mL in acetone, diluted in phosphate-buffered saline (PBS, pH 7.4)) for 10 min in the dark at room temperature, rinsed twice with PBS, and examined using epifluorescence microscopy (Nikon Eclipse Ni90, Nikon Corporation, Tokyo, Japan) with a Texas Red filter set (excitation 530–560 nm, emission 590–650 nm) [35,36]. At least 50 appressoria were analyzed per time point and strain, and the average fluorescence intensity per appressorium was calculated.

### 2.7. Western Blot Analysis of Histone Modifications

Total histones were extracted from 48 h old mycelia of wild-type, Δ*MoRph1-1*, and Δ*MoRph1-2* strains using acid extraction as previously described with modifications. Briefly, 100 mg of frozen mycelia was ground to a fine powder in liquid nitrogen and resuspended in 1 mL nuclear extraction buffer (250 mM sucrose, 10 mM Tris-HCl pH 7.5, 5 mM MgCl_2_, 1 mM phenylmethylsulfonyl fluoride (PMSF), and 1× protease inhibitor cocktail (Roche, Basel, Switzerland)). The suspension was incubated on ice for 30 min with gentle vortexing every 5 min, then centrifuged at 12,000× *g* for 15 min at 4 °C to pellet nuclei. The nuclear pellet was resuspended in 200 μL 0.4 N H_2_SO_4_ and incubated on ice for 4 h with occasional mixing to extract histones. Histones were precipitated by adding 25% trichloroacetic acid (TCA) to a final concentration of 12.5%, incubated on ice for 1 h, and centrifuged at 12,000× *g* for 15 min at 4 °C. The pellet was washed twice with acetone containing 0.1% HCl, air-dried for 15 min, and dissolved in 50 μL 4 M urea. Protein concentrations were determined using the Bradford assay (Bio-Rad, Hercules, CA, USA) with bovine serum albumin (BSA) as the standard.

Equal amounts of histone proteins (10 μg per lane) were separated by SDS-PAGE (15% polyacrylamide gel) and transferred to polyvinylidene difluoride (PVDF) membranes (Millipore, Burlington, MA, USA) at 300 mA for 90 min at 4 °C. Membranes were blocked with 5% non-fat milk in TBST (20 mM Tris-HCl pH 7.5, 150 mM NaCl, 0.1% Tween-20) for 1 h at room temperature, then incubated overnight at 4 °C with primary antibodies: anti-H3K36me3 (1:1000, Abcam, Cambridge, UK), anti-H3K4me3 (1:1000, Cell Signaling Technology, Danvers, MA, USA), anti-H3K9me3 (1:1000, Abcam), anti-H3K27me3 (1:1000, Cell Signaling Technology), anti-H3K36me1 (1:1000, Abcam), anti-H3K36me2 (1:1000, Abcam), and anti-total histone H3 (1:2000, Cell Signaling Technology). After washing three times with TBST (10 min each), membranes were incubated with horseradish peroxidase (HRP)-conjugated secondary antibodies (1:5000, goat anti-rabbit IgG, Bio-Rad) for 1 h at room temperature. Signals were detected using enhanced chemiluminescence (ECL) reagents (Millipore) and visualized with a ChemiDoc XRS+ imaging system (Bio-Rad). Band intensities were quantified using ImageJ software version 1.53t (National Institutes of Health, Bethesda, MD, USA) and normalized to total histone H3 levels. Each experiment was repeated three times independently.

### 2.8. Plant Infection and Virulence Assays

Pathogenicity was evaluated on two-week-old seedlings of susceptible rice (*Oryza sativa*) cultivar LTH (Lijiangxin Tuanheigu) and barley leaves (*Hordeum vulgare*) cultivar E9. For spray inoculation, conidial suspensions were adjusted to 1 × 10^5^ conidia/mL in 0.02% Tween-20 (Sigma-Aldrich). Spore suspensions (10 mL per pot) were sprayed onto rice seedlings. Inoculated plants were placed in a growth chamber maintained at 28 °C with 95% relative humidity for 24 h in the dark, followed by a 12 h light/12 h dark photoperiod (3000 lx) with 70% relative humidity. Disease lesions were photographed at 5 days post inoculation (dpi), and lesion areas were quantified using ImageJ software (10 leaves per strain, three replicates).

For barley leaf assays, 5 μL droplets of conidial suspension (1 × 10^5^/mL) were placed on detached leaves (5 cm in length). Plates were incubated at 28 °C with 95% relative humidity, and lesion size was measured at 5 dpi. For wound-inoculation assays on rice leaves, two-week-old LTH seedlings were wounded with a sterile needle (0.5 mm diameter) at the midrib region, and mycelial block was applied to the wound site. Inoculated plants were incubated as described above, and lesion length was measured at 5 dpi (10 leaves per strain, three replicates).

For invasive growth observation, barley leaf of one-week-old seedlings were prepared and inoculated with 50 μL conidial drops (1 × 10^5^/mL) as described [22,28]. Inoculated leaves were incubated in a moist chamber at 25 °C. At 24 and 36 hpi, the epidermal layers were excised, then invasive hyphae were observed under bright-field microscopy (Nikon Ni-U) at ×400 magnification. Penetration efficiency was calculated as (number of appressoria producing invasive hyphae/total number of appressoria) × 100%. Invasive hyphal morphology was classified as normal (branched, expanded) or abnormal (thin, unbranched, delayed elongation), with at least 100 appressoria examined per strain and time point.

### 2.9. Nuclear Integrity and DNA Damage Assays

To assess nuclear morphology under genotoxic stress, mycelia of different strains were exposed to UV-C radiation (254 nm) at a dose of 100 J/m^2^ using a germicidal lamp (20 W, Philips, Amsterdam, The Netherlands) at a distance of 30 cm. Non-irradiated mycelia served as controls. Following irradiation, samples were incubated at 28 °C for 4 h to allow DNA damage response. Nuclei were stained with Hoechst 33342 (1 μg/mL in PBS, pH 7.4; Sigma-Aldrich) for 15 min in the dark at room temperature, counterstained with Calcofluor White (50 μg/mL in PBS; Sigma-Aldrich) for 5 min to visualize cell walls, and washed three times with PBS. Samples were observed under a Nikon Eclipse Ni90 fluorescence microscope equipped with DAPI (excitation 350 nm, emission 470 nm) and UV filters.

### 2.10. Subcellular Localization

For live-cell imaging of MoRph1-GFP, conidia (1 × 10^5^ conidia/mL) and 48 h old mycelia from the MoRph1-GFP reporter strain were prepared on glass-bottom culture dishes (MatTek, Ashland, MA, USA) in liquid CM. Confocal laser scanning microscopy (CLSM) was performed using an Olympus FV3000 microscope (Tokyo, Japan) equipped with a 100× oil-immersion objective (numerical aperture 1.40). GFP fluorescence was excited at 488 nm using an argon laser, and emission was collected between 500 and 530 nm. Differential interference contrast (DIC) images were acquired simultaneously to visualize cellular morphology.

### 2.11. RNA Extraction and Transcriptome Analysis

Total RNA was extracted from 48 h old mycelia (three biological replicates for P131 and ΔMoRph1-1) using TRIzol reagent (Invitrogen, Carlsbad, CA, USA) per the manufacturer’s protocol. Approximately 100 mg frozen mycelial powder was homogenized in 1 mL TRIzol, incubated at room temperature for 5 min, mixed with 200 μL chloroform, and centrifuged at 12,000× *g* for 15 min at 4 °C. The aqueous phase was transferred to an RNase-free tube, and RNA was precipitated with 500 μL isopropanol at −20 °C for 1 h. The pellet was washed with 75% RNase-free ethanol, air-dried for 10 min, and dissolved in 30 μL RNase-free water. RNA quality was assessed via NanoDrop 2000 (A260/A280 > 1.8, A260/A230 > 2.0), 1.5% agarose gel electrophoresis (clear 28S/18S rRNA bands), and Agilent 2100 Bioanalyzer (RIN > 8.0).

RNA sequencing libraries were constructed with the NEBNext Ultra RNA Library Prep Kit for Illumina (New England Biolabs, Ipswich, MA, USA). Poly(A)-mRNA was enriched via oligo(dT) magnetic beads, fragmented into 200–300 bp, and reverse-transcribed into cDNA with random hexamers. Second-strand cDNA was synthesized with DNA polymerase I/RNase H, followed by end-repair, 3′ adenylation, and ligation to Illumina adapters. Products (200–300 bp) were size-selected via agarose gel electrophoresis, PCR-amplified (15 cycles), and purified with AMPure XP beads (Beckman Coulter, Brea, CA, USA). Libraries were sequenced on an Illumina NovaSeq 6000 platform by Novogene Co., Ltd. (Beijing, China) to generate 150 bp paired-end reads.

Raw reads were filtered with Trimmomatic (v0.39) to remove adapters, low-quality bases (Q < 20), and reads with >5% N bases. Clean reads were mapped to the *M. oryzae* P131 reference genome using HISAT2 (v2.2.1) with default parameters. Gene expression was quantified as FPKM via StringTie (v2.1.7). Differentially expressed genes (DEGs) were identified with DESeq2 (R v1.34.0) using criteria: |log_2_FC| ≥ 1 and adjusted *p* < 0.05 (Benjamini–Hochberg method). GO (MF/CC/BP) and KEGG pathway enrichment analyses were performed with clusterProfiler (R v4.2.2) using org.Mgri1.eg.db and *M. oryzae* KEGG database, respectively (*p* < 0.05). Visualization of DEGs, GO terms, and KEGG pathways was conducted with ggplot2 (R v3.4.0).

### 2.12. Quantitative Real-Time PCR (RT-qPCR) Validation

HY represents vegetative hyphae harvested from 48 h liquid CM cultures. First-strand cDNA was synthesized from 1 μg total RNA (same as RNA-seq samples) using the PrimeScript RT Reagent Kit with gDNA Eraser (Takara) per the manufacturer’s protocol to eliminate genomic DNA contamination. Quantitative PCR was performed with SYBR Green Premix Ex Taq II (Takara) on a QuantStudio 6 Flex real-time PCR system (Applied Biosystems). The 20 μL reaction mixture included 10 μL SYBR Green master mix, 0.8 μL each of 10 μM forward/reverse primers, 2 μL template cDNA (100 ng/μL), and 6.4 μL RNase-free ddH_2_O.

Thermal cycling conditions: initial denaturation at 95 °C for 30 s, followed by 40 cycles of 95 °C for 5 s and 60 °C for 34 s. Melting curve analysis (95 °C for 15 s, 60 °C for 1 min, 95 °C for 15 s) verified single-product amplification. The β-tubulin gene (MGG_00604) served as the internal reference. Relative expression was calculated via the 2^−ΔΔCt^ method [36]. All reactions included three biological replicates with technical triplicates. Statistical significance (*p* < 0.05) was assessed via Student’s *t*-test.

### 2.13. Statistical Analysis

All experiments were conducted with at least three independent biological replicates unless otherwise stated. Data are presented as means ± standard deviation (SD). Statistical significance was evaluated using Student’s *t*-test for comparisons between two groups or one-way analysis of variance (ANOVA) followed by Tukey’s honestly significant difference (HSD) post hoc test for multiple comparisons (e.g., WT vs. Δ*MoRph1* vs. MoRph1C) using GraphPad Prism 8.0 (GraphPad Software, San Diego, CA, USA) or R software (version 4.0.5). *p*-values < 0.05 were considered statistically significant. All graphs were generated using GraphPad Prism 8.0 or ggplot2 package version 3.3.5 in R software version 4.0.5.

### 2.14. Accession Numbers

The RNA-seq raw data generated in this study have been deposited in the National Center for Biotechnology Information (NCBI) Sequence Read Archive (SRA) database under the accession number PRJNA1418661.

## 3. Results

### 3.1. Deletion of MoRph1 Impairs Vegetative Growth and Asexual Development

**Figure 1:**
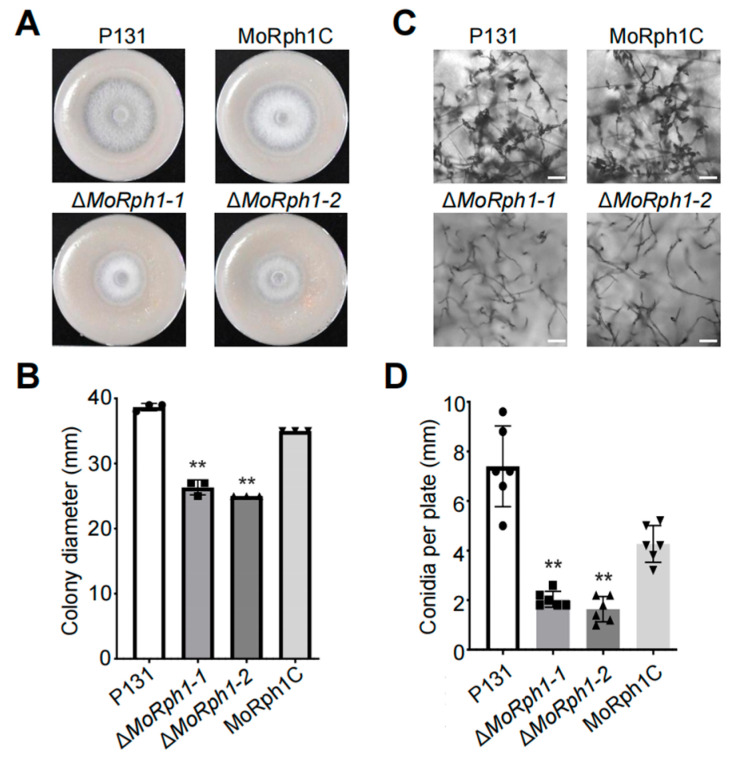
Deletion of MoRph1 impairs vegetative growth and asexual conidiation in *Magnaporthe oryzae.* (**A**) Colony morphology of the wild-type (P131), MoRph1 deletion mutants (ΔMoRph1-1, ΔMoRph1-2), and complemented strain (MoRph1C) grown on oatmeal tomato agar (OTA) at 28 °C for 7 days. (**B**) Quantitative analysis of colony diameter of the indicated strains on OTA. Data represent means ± standard deviation (SD) of three biological replicates. Asterisks indicate significant differences compared to P131 (*p* < 0.01, one-way ANOVA). (**C**) Micrographs of conidia from P131, ΔMoRph1-1, ΔMoRph1-2, and MoRph1C on OTA plates. Scale bar = 50 μm. (**D**) Conidial yield quantification of the indicated strains. Conidia were harvested from 7-day-old OTA cultures, and counts were performed using a hemocytometer. Data represent means ± SD of three biological replicates. Asterisks indicate significant differences compared to P131 (*p* < 0.01, one-way ANOVA).

To address this research objective, we generated two *MoRph1* gene deletion mutants (ΔMoRph1-1 and ΔMoRph1-2) via homologous recombination, and complete disruption of the *MoRph1* gene was verified by PCR analysis (Figure S1). We then evaluated the vegetative growth of Δ*MoRph1* on complete medium (CM) and oatmeal tomato agar (OTA), and found that the mutant exhibited a significant reduction in colony diameter compared to the wild-type strain P131 (Figure 1A,B). After 7 days of incubation, the colony size of Δ*MoRph1* was approximately 30% smaller than that of the wild type. Notably, the growth defect of Δ*MoRph1* was fully rescued in the complemented strain (MoRph1C) (Figure 1A,B), which confirms that the observed phenotypic abnormalities were specifically attributed to the loss of MoRph1 function. Intriguingly, the reduced vegetative growth and altered colony morphology of Δ*MoRph1* suggest potential defects in stress adaptation or cellular homeostasis—phenotypic features that align with the functional roles of its yeast homolog Rph1 in stress response and genome stability maintenance.

Microscopic observations further revealed that the Δ*MoRph1* mutant produced significantly fewer conidia, and the conidia that were formed displayed irregular morphologies compared to those of the wild type (Figure 1C). Quantitative analysis confirmed that the conidial yield of the mutant was reduced by approximately 75% relative to the wild-type strain (Figure 1D). As conidiation and hyphal growth are core prerequisites for *M. oryzae* to initiate host infection and spread, these results collectively demonstrate that *MoRph1* is essential for normal vegetative growth and asexual conidiation. Considering the functional role of its *S. cerevisiae* homolog Rph1 in genome stability and stress adaptation, our findings further imply that MoRph1 may be involved in regulating stress response or maintaining cellular homeostasis in *M. oryzae*, though its specific molecular mechanism and potential role in pathogenicity require further investigation.

### 3.2. MoRph1 Exhibits Stage-Specific Expression During Fungal Development and Infection

To gain insight into the temporal expression pattern of *MoRph1* during key developmental and infection stages of *M. oryzae*, we quantified its transcript levels using qRT-PCR. Total RNA was extracted from vegetative hyphae (HY), conidia (CO), appressoria at 3 h post induction (AP3H) and 12 h post induction (AP12H), as well as invasive hyphae at 18 h (IH18H), 24 h (IH24H), and 42 h post inoculation (IH42H) (Figure S2). The results showed that MoRph1 exhibited stage-specific expression across the tested developmental stages. Notably, MoRph1 expression showed stage-dependent variation, with relatively higher transcript levels detected in HY, CO, and IH42H (Figure S2). This expression pattern aligns with the phenotypic defects observed in the ΔMoRph1 mutant: high expression in mycelia and conidia correlates with the mutant’s impaired vegetative growth and conidiation, while elevated expression in late-stage invasive hyphae (IH42H) suggests a potential role in sustaining invasive growth and completing the infection cycle. These data collectively indicate that MoRph1 is dynamically regulated during *M. oryzae* development and infection, with high expression in stages critical for fungal growth, sporulation, and late-stage host colonization.

### 3.3. MoRph1 Is Required for Full Pathogenicity and Host Invasion

**Figure 2:**
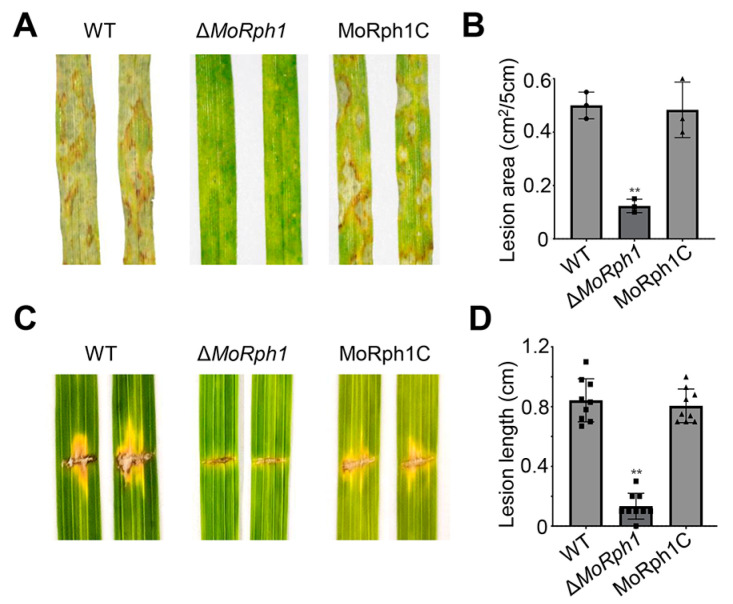
MoRph1 is required for full pathogenicity of *M. oryzae* on host plants. (**A**) Pathogenicity assays on barley leaves at 4 days post inoculation (dpi). The Δ*MoRph1* mutant induces sparse, small necrotic lesions, while WT and MoRph1C produce extensive typical rice blast lesions. (**B**) Quantitative analysis of lesion area on rice leaves. Data represent means ± SD of 10 leaves per strain (three biological replicates). Asterisks indicate significant differences compared to WT (*p* < 0.01, one-way ANOVA). (**C**) Wound-inoculation assays on rice leaves. Lesion expansion is severely impaired in Δ*MoRph1*. (**D**) Quantitative analysis of lesion length on wound-inoculated rice leaves. Data represent means ± SD of 8 leaves per strain. Asterisks indicate significant differences compared to P131 (*p* < 0.01, one-way ANOVA).

To systematically assess the role of MoRph1 in fungal virulence, we performed spray-inoculation assays on seedlings of the susceptible rice cultivar LTH and detached leaves of barley. At 5 days post inoculation (dpi), the Δ*MoRph1* mutant induced only sparse, small necrotic lesions on both hosts, whereas the wild-type (P131) and complemented (MoRph1C) strains produced extensive, typical rice blast lesions (Figure 2A). Quantitative measurement of lesion size further confirmed a dramatic attenuation of virulence in the mutant relative to the control strains (Figure 2B). To further validate the virulence defect, we conducted wound-inoculation assays on rice leaves, which simulate the scenario of pathogen invasion through host tissue wounds. The results showed that lesion expansion was severely impaired in the ΔMoRph1 mutant; statistical analysis of lesion length consistently demonstrated a significant reduction in the mutant compared to the wild-type and complemented strains (Figure 2C,D). Taken together, these virulence assay results clearly demonstrate that MoRph1 is indispensable for the full pathogenicity of *M. oryzae* on its host plants.

### 3.4. MoRph1 Regulates Appressorial Penetration and Invasive Growth

**Figure 3:**
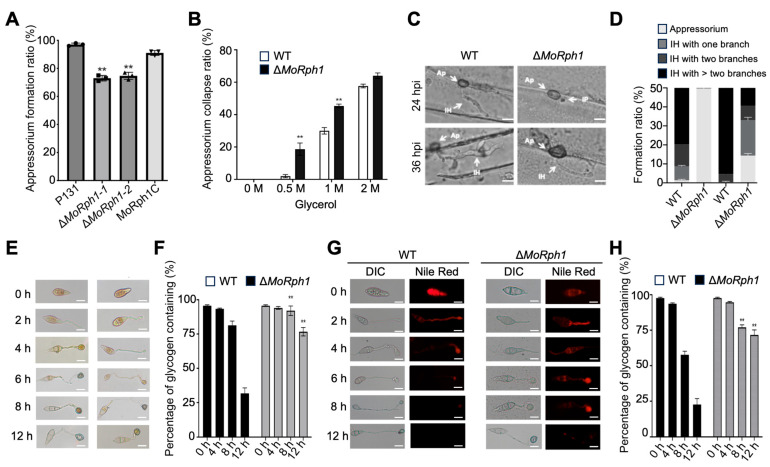
MoRph1 regulates appressorial function, host penetration, and energy reserve mobilization. (**A**) Appressorium formation rate of P131, Δ*MoRph1*, and MoRph1C at 12 h post induction (hpi) on hydrophobic glass coverslips. Data represent means ± SD of three biological replicates (≥100 conidia counted per replicate). Asterisks indicate significant differences compared to P131 (*p* < 0.01, one-way ANOVA). (**B**) Cytorrhysis assay to measure appressorial turgor pressure. Mature appressoria (24 hpi) were treated with 0, 0.5, 1 and 2 M glycerol for 10 min, and collapsed appressoria were counted. Data represent means ± SD of three biological replicates (≥100 appressoria counted per replicate). Asterisks indicate significant differences compared to P131 (*p* < 0.01, one-way ANOVA). (**C**) Barley leaf penetration and invasive growth assays. Epidermal layers were observed at 24 hpi and 36 hpi after inoculation. Scale bar = 10 μm. (**D**) Formation ratios of appressorium and invasive hypha (IH) of the indicated strains. Data represent means ± SD of three biological replicates (≥100 appressoria counted per replicate). Asterisks indicate significant differences compared to P131 (*p* < 0.01, one-way ANOVA). (**E**) Glycogen dynamics during appressorial development. KI/I_2_ staining of glycogen (dark brown deposits) at indicated time points. Scale bar = 10 μm. (**F**) Percentage of glycogen containing in conidium or appressorium. Data represent means ± SD of three fields of view (≥50 appressoria per field). (**G**) Lipid droplet dynamics during appressorial development. Nile Red staining of neutral lipids (red fluorescence) at indicated time points. Scale bar = 10 μm. (**H**) Percentage of lipid droplet containing in conidium or appressorium. Data represent means ± SD of three fields of view (≥50 appressoria per field). Asterisks in (**F**,**H**) indicate significant differences between Δ*MoRph1* and P131 at the same time point (*p* < 0.05, Student’s *t*-test).

Appressorium formation is a pivotal initial step for *M. oryzae* to invade host tissues. We first evaluated appressorium development of the Δ*MoRph1* mutant, and found that at 12 h post induction (hpi), only approximately 50% of germinated Δ*MoRph1* conidia formed mature appressoria, whereas the wild-type strain (P131) achieved an appressorium formation rate of approximately 90% (Figure 3A), indicating that MoRph1 is required for efficient appressorium maturation.

Since appressorial turgor pressure serves as the core driving force for breaching the host cuticle, we further investigated whether the mutant’s impaired penetration capacity was associated with defective turgor generation. Cytorrhysis assays using hyperosmotic glycerol solutions revealed that Δ*MoRph1* appressoria were significantly more susceptible to collapse than those of the wild type. When treated with 2 M glycerol, over 80% of mutant appressoria collapsed, compared to only approximately 30% of wild-type appressoria (Figure 3B), confirming that MoRph1 deficiency leads to defective appressorial turgor pressure.

To comprehensively characterize the host invasion defects of Δ*MoRph1*, we performed barley leaf penetration assays, which enable detailed observation of the infection process at the cellular level. Although Δ*MoRph1* conidia could germinate and form appressoria to a certain extent, its efficiency in penetrating the host epidermis was drastically reduced. At 24 hpi, fewer than 25% of Δ*MoRph1* appressoria successfully penetrated the barley epidermal layer, whereas the penetration rate of wild-type appressoria exceeded 70% (Figure 3C). Moreover, even when penetration was achieved, the invasive hyphae of Δ*MoRph1* exhibited obvious abnormalities: compared to the wild-type and complemented (MoRph1C) strains, mutant invasive hyphae were noticeably thinner, displayed delayed elongation, and failed to form normal branched hyphal structures (Figure 3C,D).

Collectively, these findings demonstrate that MoRph1 (MGG_09186) is indispensable for multiple key steps during *M. oryzae* host invasion, including efficient appressorium formation and functionality, successful host cuticle penetration, and subsequent establishment of invasive hyphal growth—all of which are critical for the pathogen to complete its infection cycle and exert full pathogenicity.

### 3.5. MoRph1 Regulates Appressorial Energy Mobilization

Given the defective appressorial turgor pressure observed in the ΔMoRph1 mutant, we further investigated whether this defect stemmed from impaired catabolism of energy reserves—an essential process for turgor generation during appressorium maturation. To this end, we monitored the dynamics of glycogen and lipid droplets, two key energy storage molecules, throughout appressorium development.

Glycogen dynamics were assessed using potassium iodide (KI/I_2_) staining, a specific method for glycogen visualization. In the wild-type strain (P131), glycogen reserves were rapidly depleted during the critical maturation stage (4 to 8 hpi), as evidenced by the gradual fading of staining intensity (Figure 3E,F). In sharp contrast, the Δ*MoRph1* mutant retained substantial glycogen levels even at 12 hpi, with no obvious reduction in staining signal compared to earlier time points (Figure 3E,F). This result indicates that MoRph1 is required for the efficient mobilization of glycogen reserves during appressorium maturation.

Lipid droplet dynamics were analyzed via Nile Red staining, which specifically labels neutral lipids. Consistent with the glycogen mobilization pattern, wild-type appressoria showed rapid depletion of lipid droplets between 4 and 8 hpi, as reflected by the marked decrease in fluorescent signal (Figure 3G,H). However, the Δ*MoRph1* mutant failed to efficiently break down lipid droplets; significant levels of lipid droplets persisted in mutant appressoria even at 12 hpi (Figure 3G,H), as indicated by the sustained strong fluorescent signal.

Collectively, these data demonstrate that MoRph1 is indispensable for the efficient mobilization of both glycogen and lipid reserves during appressorium maturation. The impaired catabolism of these energy stores in the Δ*MoRph1* mutant directly contributes to the defective turgor pressure generation, which ultimately compromises the pathogen’s ability to penetrate host tissues.

### 3.6. MoRph1 Functions as an H3K36me3-Specific Histone Demethylase

**Figure 4:**
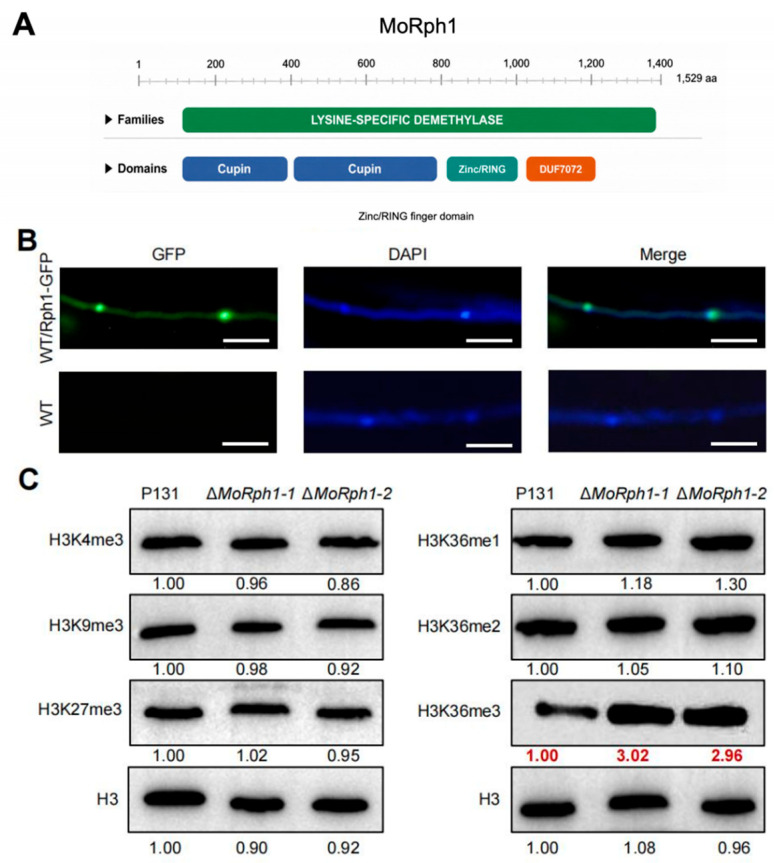
MoRph1 is an H3K36me3-specific histone demethylase localized to the nucleus. (**A**) Schematic diagram of MoRph1 functional domains predicted by InterPro. Domains include lysine-specific demethylase domain (containing two cupin domains), Zinc/RING finger domain, C3HC4-type zinc finger domain, and domain of unknown function (DUF7072). (**B**) Subcellular localization of MoRph1-GFP. Confocal laser scanning microscopy (CLSM) images showing GFP fluorescence (green), DAPI nuclear staining (blue), merged signals images of mycelial cells from the MoRph1-GFP reporter strain (WT/MoRph1-GFP) and wild-type P131 control (WT). Scale bar = 20 μm. (**C**) Western blot analysis of histone methylation marks in P131, ΔMoRph1-1, and ΔMoRph1-2. Total histones were probed with antibodies against H3K36me3, H3K4me3, H3K9me3, H3K27me3, H3K36me1, H3K36me2, and total histone H3 (loading control). Band intensities were quantified using ImageJ and normalized to total H3 levels (bottom panel).

Sequence analysis via InterPro (https://www.ebi.ac.uk/interpro/, accessed on 4 May 2026) revealed that MoRph1 contains multiple functional domains, including a lysine-specific demethylase domain (encompassing two cupin domains), a Zinc/RING finger domain, a C3HC4-type zinc finger domain, and a domain of unknown function (DUF7072). Among these, the lysine-specific demethylase domain is a signature feature of Fe(II)- and α-ketoglutarate-dependent histone demethylases, which is indispensable for their catalytic activity (Figure 4A).

To examine the subcellular localization of MoRph1, we constructed a MoRph1-GFP fusion expression vector driven by the constitutive RP27 promoter and transformed it into the wild-type strain P131. The MoRph1-GFP strain exhibits normal colony size and morphology comparable to the wild-type strain P131 (Figure S3). Confocal laser scanning microscopy (CLSM) showed that GFP fluorescence was predominantly detected in nuclear regions of mycelial cells (Figure 4B), consistent with a nuclear localization pattern.

To examine whether MoRph1 affects histone methylation status, we performed Western blot analysis using antibodies against H3K4me3, H3K9me3, H3K27me3, H3K36me1, H3K36me2, and H3K36me3. Compared with the wild-type strain P131, the ΔMoRph1 mutant showed an increased H3K36me3 signal (Figure 4C). In contrast, no obvious differences were detected in the levels of H3K4me3, H3K9me3, H3K27me3, H3K36me1, or H3K36me2 between the mutant and the wild type (Figure 4C). These results support an association between MoRph1 and H3K36me3 demethylation in *M. oryzae*.

H3K36me3 is a histone mark associated with transcriptional elongation and chromatin organization in eukaryotes. The altered H3K36me3 level observed in the ΔMoRph1 mutant suggests that MoRph1 contributes to epigenetic regulation of gene expression in *M. oryzae*. This finding is consistent with the developmental and pathogenicity-related defects observed in the mutant, including impaired vegetative growth, reduced conidiation, defective appressorial function, and compromised host penetration.

### 3.7. MoRph1 Function as a Master Epigenetic Regulator

**Figure 5:**
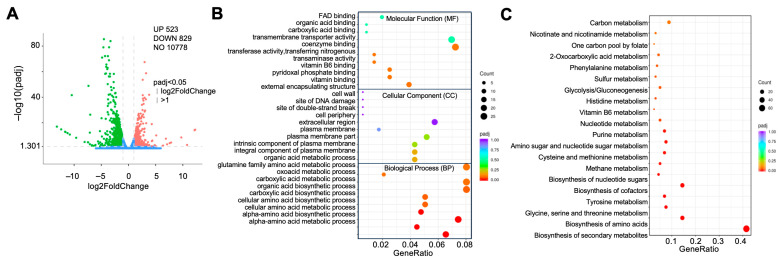
Transcriptome analysis reveals MoRph1-regulated genes involved in core biological processes. (**A**) Volcano plot of differentially expressed genes (DEGs) between ΔMoRph1-1 and P131. DEGs were identified with log_2_ fold change (log_2_FC) ≥ ±1 and adjusted *p* < 0.05 (DESeq2). Red dots represent upregulated genes (*n* = 523), green dots represent downregulated genes (*n* = 829), and gray dots represent non-differentially expressed genes (*n* = 10,778). (**B**) Gene Ontology (GO) enrichment analysis of DEGs. Significantly enriched GO terms (adjusted *p* < 0.05) are categorized into Molecular Function (MF), Cellular Component (CC), and Biological Process (BP). The top 10 enriched terms in each category are shown. The size of each bubble represents the number of DEGs in the pathway; the color intensity indicates the adjusted *p*-value. (**C**) Kyoto Encyclopedia of Genes and Genomes (KEGG) pathway enrichment analysis of DEGs. Significantly enriched pathways (adjusted *p* < 0.05) are grouped into four functional clusters: Carbon and Energy Metabolism, Amino Acid and Vitamin Metabolism, Nucleotide and Nucleotide Sugar Metabolism, and Coenzyme and Secondary Metabolism. The size of each bubble represents the number of DEGs in the pathway; the color intensity indicates the adjusted *p*-value.

To examine the transcriptional changes associated with MoRph1 deletion, we performed RNA sequencing (RNA-seq) analysis to compare the global gene expression profiles between the ΔMoRph1 mutant and the wild-type strain P131. Using stringent screening criteria (log_2_ fold change ≥ ±1 and adjusted *p*-value < 0.05), a total of 1352 differentially expressed genes (DEGs) were identified, including 523 upregulated and 829 downregulated transcripts in the ΔMoRph1 mutant (Figure 5A, Table S1). These results indicate that loss of MoRph1 is associated with broad transcriptional changes in *M. oryzae*.

To dissect the biological functions of the identified DEGs, we performed GO enrichment analysis, which classified the DEGs into three major categories: Molecular Function (MF), Cellular Component (CC), and Biological Process (BP) (Figure 5B).

The DEGs were predominantly enriched in MF terms associated with binding activity, transporter activity, and transferase activity. Specifically, enrichment of FAD binding, coenzyme binding, vitamin B6 binding, and pyridoxal phosphate binding suggests that MoRph1 regulates the expression of genes encoding enzymes involved in redox reactions and coenzyme-dependent metabolic processes—key pathways for energy metabolism and biosynthesis of essential metabolites. For instance, vitamin B6 (pyridoxal phosphate as its active form) is critical for amino acid transamination and metabolism, which is consistent with the impaired energy mobilization (glycogen and lipid droplet catabolism) observed in Δ*MoRph1* appressoria. Enrichment of transmembrane transporter activity indicates that MoRph1 modulates the expression of genes responsible for substance transport across cell membranes, which may affect nutrient uptake, ion homeostasis, and secretion of virulence factors—all of which are essential for vegetative growth and host–pathogen interaction.

In the CC category, the DEGs were significantly enriched in terms related to the cell wall, plasma membrane, extracellular region, and DNA damage sites. Enrichment of cell wall, external encapsulating structure, and plasma membrane-related terms (e.g., intrinsic/integral component of plasma membrane) is highly consistent with the altered colony morphology and potential cell wall defects of the Δ*MoRph1* mutant. These DEGs may include genes encoding cell wall synthesis enzymes or membrane transporters, whose dysregulation would lead to impaired cell wall integrity and membrane function—directly contributing to defective vegetative growth and appressorial penetration. Notably, enrichment of terms such as “site of DNA damage” and “site of double-strand break” aligns with the functional analogy of MoRph1 to *S. cerevisiae* Rph1 (a regulator of genome stability and DNA repair). This suggests that MoRph1 may regulate the expression of DNA repair-related genes, and its deletion leads to compromised genome stability in *M. oryzae*, which could further exacerbate phenotypic defects under stress conditions.

The BP category enrichment results showed that the DEGs were mainly involved in organic acid and amino acid metabolic/biosynthetic processes, including glutamine family amino acid metabolism, α-amino acid metabolism, and carboxylic acid metabolism. These processes are central to energy production (e.g., via glycolysis and TCA cycle) and biosynthesis of essential biomolecules (proteins, nucleotides), which are indispensable for fungal vegetative growth, conidiation, and appressorial maturation. Further refinement of BP enrichment analysis (Figure 5C) revealed additional key processes: DNA replication, mismatch repair, homologous recombination (linking to genome stability, consistent with Rph1 homolog function), oxidative stress response, and chromatin organization (directly reflecting MoRph1’s role as a histone demethylase). Collectively, these enriched BP terms indicate that MoRph1 regulates a suite of biological processes core to *M. oryzae*’s growth, development, stress adaptation, and pathogenicity, explaining the diverse phenotypic abnormalities of the Δ*MoRph1* mutant at the transcriptional level.

To further clarify the metabolic and signaling pathways modulated by MoRph1, we performed KEGG pathway enrichment analysis (Figure 5C). The results showed that the DEGs were significantly enriched in multiple metabolic pathways, which can be classified into four major functional clusters: Carbon and Energy Metabolism Pathways, Amino Acid and Vitamin Metabolism Pathways, Nucleotide and Nucleotide Sugar Metabolism Pathways and Coenzyme and Secondary Metabolism Pathways. Carbon and energy metabolism pathways include Carbon metabolism, Glycolysis/Gluconeogenesis, 2-Oxocarboxylic acid metabolism, and Methane metabolism. Dysregulation of these pathways in the Δ*MoRph1* mutant directly explains its defective energy mobilization and reduced growth rate. Amino acid and vitamin metabolism pathways such as Phenylalanine metabolism, Histidine metabolism, Cysteine and methionine metabolism, Glycine, serine and threonine metabolism, Biosynthesis of amino acids, and Vitamin B6 metabolism were significantly enriched. Amino acid metabolism is not only critical for protein synthesis but also provides precursors for cell wall components and secondary metabolites. Vitamin B6 metabolism, consistent with the MF enrichment result, is essential for amino acid transamination and redox reactions. Dysregulation of these pathways in the Δ*MoRph1* mutant would impair cellular homeostasis, growth, and pathogenicity-related processes. Nucleotide and nucleotide sugar metabolism pathways include Nucleotide metabolism, Purine metabolism, Amino sugar and nucleotide sugar metabolism, and Biosynthesis of nucleotide sugars. Nucleotide metabolism is fundamental for DNA replication, repair, and RNA transcription, processes closely regulated by histone methylation. Amino sugar and nucleotide sugar metabolism provides precursors for cell wall biosynthesis, which aligns with the CC enrichment of cell wall-related terms and the Δ*MoRph1* mutant’s potential cell wall defects. Coenzyme and secondary metabolism pathways such as Nicotinate and nicotinamide metabolism, One carbon pool by folate, Biosynthesis of cofactors, and Biosynthesis of secondary metabolites were enriched. Dysregulation of these pathways further supports the role of MoRph1 in modulating stress adaptation and pathogenicity.

Collectively, the RNA-seq and enrichment analysis results demonstrate that MoRph1, as a master epigenetic regulator, modulates the transcription of a broad range of genes involved in energy metabolism, cell wall biosynthesis, genome stability, amino acid metabolism, and stress response. These regulatory effects are mediated by its H3K36me3-specific demethylase activity, and dysregulation of these downstream genes in the Δ*MoRph1* mutant is the molecular basis for its diverse phenotypic defects in growth, development, and pathogenicity. This further confirms that MoRph1 plays an indispensable role in integrating epigenetic regulation with key biological processes to support *M. oryzae*’s survival and infection.

### 3.8. MoRph1 Regulates DNA Damage Response Genes

Given the enrichment of DNA replication, repair, and genome stability-related pathways in the transcriptome analysis, we further examined whether MoRph1 is involved in the DNA damage response (DDR) in *M. oryzae*. To this end, we evaluated nuclear morphology in hyphal cells following UV-induced DNA damage, as nuclear integrity is closely associated with genome stability under genotoxic stress.

**Figure 6:**
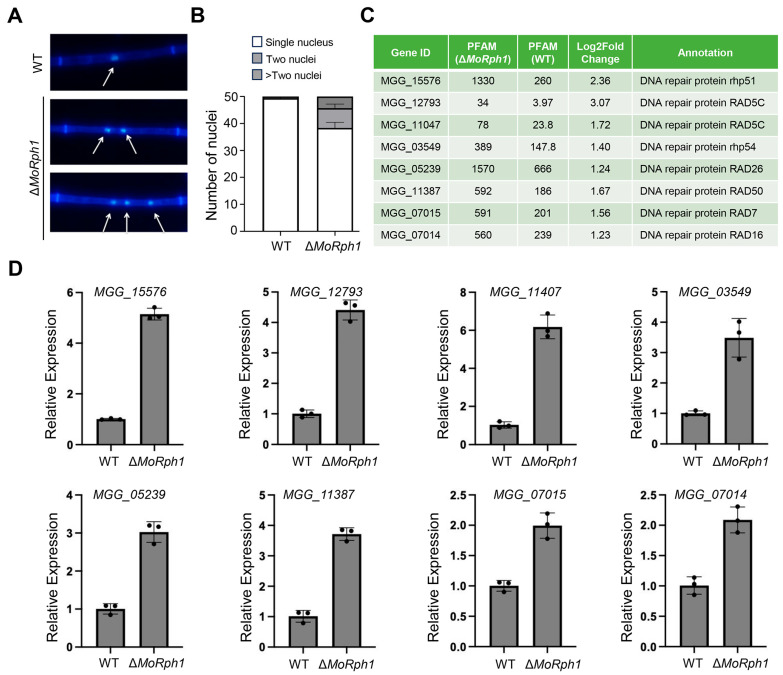
MoRph1 regulates DNA damage response (DDR) and genome stability. (**A**) Nuclear morphology of P131 and ΔMoRph1-1 hyphal cells after UV-C treatment (100 J/m^2^) and Hoechst 33342 staining. Nuclei are indicated by white arrows. (**B**) Percentage of hyphal cells containing one or multiple nuclear signals in WT and Δ*MoRph1*. Data represent means ± SD of three biological replicates (≥100 hyphal cells cell counted per replicate). (**C**) DDR-related DEGs identified by RNA-seq. (**D**) RT-qPCR validation of selected DDR-related DEGs. The β-tubulin gene (*MGG_00604*) was used as an internal reference. Relative expression levels were calculated using the 2^−ΔΔCt^ method.

Hoechst 33342 staining revealed that ΔMoRph1 mutant hyphal cells exhibited a significantly higher frequency of abnormal nuclear morphology than the wild-type strain P131 after UV treatment (Figure 6A). Specifically, over 40% of ΔMoRph1 hyphal cells contained multiple nuclear signals, whereas only approximately 10% of wild-type hyphal cells showed similar changes (Figure 6B). These results suggest that MoRph1 contributes to maintaining nuclear integrity under genotoxic stress conditions.

To further decipher the molecular mechanism underlying MoRph1-mediated genome stability regulation, we analyzed the RNA-seq data for differentially expressed genes (DEGs) involved in the DNA damage response pathway. Among the identified DEGs, several key components of the DDR pathway were found to be transcriptionally dysregulated in the Δ*MoRph1* mutant, including core DNA repair proteins: MGG_15576 (RAD51), MGG_12793 (RAD5C), MGG_11047 (RAD16), MGG_03549 (RAD54), MGG_05239 (RAD26), MGG_11387 (RAD50), MGG_07015 (RAD7), and MGG_07014 (RAD16) (MGG_07014 and MGG_11047 both encode RAD16 homologs, likely functionally redundant isoforms). These RAD family proteins are critical for homologous recombination repair, nucleotide excision repair, and DNA double-strand break repair, core processes for maintaining genome integrity. The coordinated transcriptional dysregulation of these DDR-related genes in the Δ*MoRph1* mutant further implicates MoRph1 in the transcriptional control of genomic stress response networks (Figure 6C).

To validate the RNA-seq results, we performed reverse transcription-quantitative PCR (RT-qPCR) analysis on representative DDR-related DEGs. The RT-qPCR results showed expression trends consistent with the RNA-seq data (Figure 6D), supporting the reliability of the transcriptome analysis.

Collectively, these results indicate that MoRph1 is associated with regulation of DDR-related gene expression and maintenance of nuclear integrity under genotoxic stress. The loss of MoRph1 was accompanied by altered H3K36me3 levels, transcriptional changes in DDR-related genes, and abnormal nuclear morphology after UV treatment. These findings are consistent with the diverse phenotypic defects observed in the ΔMoRph1 mutant and support a role for MoRph1 in linking epigenetic regulation with genome stability in *M. oryzae*.

## 4. Discussion

In this study, we identified and characterized MoRph1 as a critical epigenetic regulator that integrates chromatin remodeling with DNA damage repair and metabolic reprogramming during fungal pathogenesis. Our findings reveal that MoRph1-dependent H3K36me3 demethylation serves as a molecular nexus coordinating developmental transitions, energy mobilization, and genome stability maintenance in *M. oryzae*. These results not only expand our understanding of histone modification dynamics in phytopathogenic fungi but also establish a previously unrecognized link between epigenetic regulation and infection-related metabolic adaptation.

### 4.1. MoRph1 Bridges Epigenetic Regulation and DNA Damage Response

The identification of MoRph1 as a functional H3K36me3-specific demethylase represents a significant advancement in our understanding of chromatin-mediated stress responses in plant-pathogenic fungi. While H3K36me3 demethylases have been characterized in model organisms such as *S. cerevisiae* (Rph1 and Jhd1) [15,16,17], our study provides the first evidence that this epigenetic mechanism is conserved and essential in *M. oryzae*. The observation that MoRph1 loss results in H3K36me3 accumulation and concomitant dysregulation of DNA repair genes (*RAD51*, *RAD5C*, *RAD16*, *RAD54*, *RAD26*, *RAD50*, *RAD7*, and *RAD16*) suggests that, analogous to yeast Rph1, MoRph1 functions as a transcriptional modulator of the DNA damage response pathway.

However, distinct from the yeast paradigm where Rph1 primarily represses photolyase gene *PHR1* under non-stress conditions [16,17,18], MoRph1 appears to exert broader transcriptional control over multiple DNA repair mechanisms, including homologous recombination and base excision repair. This expanded regulatory scope likely reflects the specialized lifestyle of *M. oryzae*, which must maintain genome integrity while encountering host-derived oxidative bursts and DNA-damaging agents during invasive growth [19,20]. The elevated nuclear fragmentation observed in Δ*MoRph1* following UV exposure (>40% versus ~10% in wild-type) underscores the critical requirement for active H3K36me3 demethylation in resolving genotoxic stress, potentially through the regulation of histone exchange mechanisms and chromatin accessibility at DNA break sites.

### 4.2. Epigenetic Control of Energy Metabolism and Infection Structure Function

A particularly novel aspect of our findings is the revelation that MoRph1-mediated epigenetic regulation directly governs energy reserve mobilization during appressorial development. The delayed catabolism of glycogen and lipid bodies in Δ*MoRph1* appressoria, despite the morphological formation of infection structures, establishes a direct causal link between H3K36me3 dynamics and metabolic flux required for turgor generation. This observation extends the canonical role of H3K36me3 in transcriptional elongation to include the regulation of catabolic gene networks essential for host penetration.

The resulting defect in appressorial turgor pressure, evidenced by >80% collapse at 2 M glycerol compared to ~30% in wild-type, demonstrates that epigenetic regulation constitutes a critical control layer for the mechanical force generation required for pathogenicity. We propose that MoRph1 facilitates the transcriptional switch from energy storage to energy utilization by erasing H3K36me3 marks at specific loci encoding catabolic enzymes and cell wall remodeling factors. This epigenetic-metabolic coupling represents a specialized adaptation that enables rapid appressorial maturation and host cuticle breach, distinguishing *M. oryzae* from hemibiotrophic or necrotrophic pathogens that may rely on different nutritional strategies.

### 4.3. Nuclear Integrity and Genome Stability Maintenance

Our observation of increased nuclear abnormalities in Δ*MoRph1* under DNA damage conditions introduces another dimension to MoRph1 function: the maintenance of nuclear architecture during genotoxic stress. The formation of micronuclei and nuclear fragmentation in the mutant suggests that H3K36me3 demethylation is required for proper DNA double-strand break repair and chromosome segregation. This function may involve the recruitment of DNA repair machinery to damage sites or the resolution of recombination intermediates through chromatin remodeling.

The nuclear localization of MoRph1, consistent with its role as a chromatin-modifying enzyme, positions it at the center of a stress-responsive regulatory hub. Unlike earlier developmental stages where we could not detect fluorescent fusion proteins in conidia, the functional evidence from DNA damage assays clearly establishes nuclear activity. This temporal regulation of MoRph1 function—potentially activated during specific cell cycle checkpoints or in response to DNA damage signals—warrants further investigation into its interaction partners, including potential kinases analogous to Rad53 in yeast that might mediate stress-induced activation [16].

### 4.4. Implications and Future Perspectives

The present study demonstrates that MoRph1 is involved in multiple biological processes in *Magnaporthe oryzae*, including vegetative growth, conidiation, appressorium-mediated infection, and the response to DNA damage stress. These findings expand current understanding of how histone demethylation contributes to fungal development and pathogenicity.

At the same time, the broader significance of MoRph1 should not be overstated. Although the mutant phenotypes indicate that MoRph1 is important for full virulence, the present work did not investigate inhibitor sensitivity, structural determinants of enzymatic activity, or possible selectivity relative to homologous proteins in other organisms. Accordingly, whether MoRph1 can serve as a tractable antifungal target remains unclear and requires further study.

Future work should combine genetic, biochemical, and structural approaches to identify the direct targets and molecular partners of MoRph1 and to clarify how this histone demethylase regulates infection-associated transcriptional programs. These efforts will provide a more complete framework for understanding the contribution of epigenetic regulation to rice blast fungus biology.

## Data Availability

The original contributions presented in the study are included in the article/Supplementary Materials, further inquiries can be directed to the corresponding author.

## Supplementary Materials

The following supporting information can be downloaded at: https://www.mdpi.com/article/10.3390/jof12050338/s1, Figure S1: PCR verification of *MoRph1* gene deletion and complementation; Figure S2: Transcript profile of *MoRph1* at different developmental stages; Figure S3: Colony morphology and growth of the MoRph1–GFP fusion strain compared with the wild-type strain P131; Table S1: List of differentially expressed genes identified by RNA-seq analysis.

## References

[1] Savary S., Willocquet L., Pethybridge S.J., Esker P., McRoberts N., Nelson A. (2019). The global burden of pathogens and pests on major food crops. Nat. Ecol. Evol..

[2] Ibrahim E., Luo J., Ahmed T., Wu W., Yan C., Li B. (2020). Biosynthesis of silver nanoparticles using onion endophytic bacterium and its antifungal activity against rice pathogen *Magnaporthe oryzae*. J. Fungi.

[3] Longya A., Talumphai S., Jantasuriyarat C. (2020). Morphological characterization and genetic diversity of rice blast fungus, pyricularia oryzae, from thailand using ISSR and SRAP markers. J. Fungi.

[4] Hamer J.E., Howard R.J., Chumley F.G., Valent B. (1988). A mechanism for surface attachment in spores of a plant pathogenic fungus. Science.

[5] Ta Bui L., Nguyen P.H. (2023). Assessment of rice yield and economic losses caused by ground-level O_3_ exposure in the Mekong delta region, Vietnam. Heliyon.

[6] Xue M., Yang J., Li Z., Hu S., Yao N., Dean R.A., Zhao W., Shen M., Zhang H., Li C. (2012). Comparative analysis of the genomes of two field isolates of the rice blast fungus *Magnaporthe oryzae*. PLoS Genet..

[7] Dean R.A., Talbot N.J., Ebbole D.J., Farman M.L., Mitchell T.K., Orbach M.J., Thon M., Kulkarni R., Xu J.R., Pan H. (2005). The genome sequence of the rice blast fungus *Magnaporthe grisea*. Nature.

[8] Couch B.C., Kohn L.M. (2002). A multilocus gene genealogy concordant with host preference indicates segregation of a new species, *Magnaporthe oryzae*, from *M. grisea*. Mycologia.

[9] Yang J., Zhao X., Sun J., Kang Z., Ding S., Xu J.R., Peng Y.L. (2010). A novel protein Com1 is required for normal conidium morphology and full virulence in *Magnaporthe oryzae*. Mol. Plant-Microbe Interact..

[10] Kong L.A., Yang J., Li G.T., Qi L.L., Zhang Y.J., Wang C.F., Zhao W.S., Xu J.R., Peng Y.L. (2012). Different chitin synthase genes are required for various developmental and plant infection processes in the rice blast fungus *Magnaporthe oryzae*. PLoS Pathog..

[11] Xu X.W., Zhao R., Xu X.Z., Tang L., Shi W., Chen D., Peng J.B., Bhadauria V., Zhao W.S., Yang J. (2022). Mosnf5 regulates fungal virulence, growth, and conidiation in *Magnaporthe oryzae*. J. Fungi.

[12] Huh A., Dubey A., Kim S., Jeon J., Lee Y.H. (2017). *MoJMJ1*, encoding a histone demethylase containing Jmjc domain, is required for pathogenic development of the rice blast fungus, *Magnaporthe oryzae*. Plant Pathol. J..

[13] Cao Z., Yin Y., Sun X., Han J., Sun Q.P., Lu M., Pan J., Wang W. (2016). An Ash1-like protein MoKMT2H null mutant is delayed for conidium germination and pathogenesis in *Magnaporthe oryzae*. BioMed Res. Int..

[14] Berry W.L., Janknecht R. (2013). KDM4/JMJD2 histone demethylases: Epigenetic regulators in cancer cells. Cancer Res..

[15] Klose R.J., Gardner K.E., Liang G., Erdjument-Bromage H., Tempst P., Zhang Y. (2007). Demethylation of histone H3K36 and H3K9 by Rph1: A vestige of an H3K9 methylation system in *Saccharomyces cerevisiae*?. Mol. Cell. Biol..

[16] Kim E.M., Jang Y.K., Park S.D. (2002). Phosphorylation of Rph1, a damage-responsive repressor of *PHR1* in *Saccharomyces cerevisiae*, is dependent upon Rad53 kinase. Nucleic Acids Res..

[17] Jang Y.K., Wang L., Sancar G.B. (1999). RPH1 and GIS1 are damage-responsive repressors of PHR1. Mol. Cell. Biol..

[18] Chatterjee N., Walker G.C. (2017). Mechanisms of DNA damage, repair, and mutagenesis. Environ. Mol. Mutagen..

[19] Jackson S.P., Bartek J. (2009). The DNA-damage response in human biology and disease. Nature.

[20] Sancar A., Lindsey-Boltz L.A., Unsal-Kacmaz K., Linn S. (2004). Molecular mechanisms of mammalian DNA repair and the DNA damage checkpoints. Annu. Rev. Biochem..

[21] Williams A.B., Schumacher B. (2016). P53 in the DNA-damage-repair process. Cold Spring Harb. Perspect. Med..

[22] Peng Y.-L., Shishiyama J. (1988). Temporal sequence of cytological events in rice leaves infected with *Pyricularia oryzae*. Can. J. Bot..

[23] Gao X., Yin C., Liu X., Peng J., Chen D., He D., Shi W., Zhao W., Yang J., Peng Y.-L. (2019). A glycine-rich protein MoGrp1 functions as a novel splicing factor to regulate fungal virulence and growth in *Magnaporthe oryzae*. Phytopathol. Res..

[24] Shi W., Yang J., Chen D., Yin C., Zhang H., Xu X., Pan X., Wang R., Fei L., Li M. (2022). The rice blast fungus SR protein 1 regulates alternative splicing with unique mechanisms. PLoS Pathog..

[25] Li Z., Yang J., Peng J., Cheng Z., Liu X., Zhang Z., Bhadauria V., Zhao W., Peng Y.L. (2021). Transcriptional landscapes of long non-coding RNAs and alternative splicing in *Pyricularia oryzae* revealed by RNA-Seq. Front. Plant Sci..

[26] Chen X.L., Shi T., Yang J., Shi W., Gao X., Chen D., Xu X., Xu J.R., Talbot N.J., Peng Y.L. (2014). N-glycosylation of effector proteins by an alpha-1,3-mannosyltransferase is required for the rice blast fungus to evade host innate immunity. Plant Cell.

[27] Wang R.-J., Cui D., Zhao R., Jin Y., Zeng W., Yang Y., Qi L., Xiang L., Peng Y.-L. (2023). The regulatory subunit MoB56 of PP2A phosphatase regulates pathogenicity, growth and development in a protein complex with the atypical catalytic subunit Ppg1 in the rice blast fungus *Magnaporthe oryzae*. Phytopathol. Res..

[28] Liu D., Lun Z., Liu N., Yuan G., Wang X., Li S., Peng Y.L., Lu X. (2023). Identification and characterization of novel candidate effector proteins from *Magnaporthe oryzae*. J. Fungi.

[29] Yamada M., Kiyosawa S., Yamaguchi T., Hirano T., Kobayashi T., Kushibuchi K., Watanabe S. (1976). Proposal of a new method for differentiating races of *Pyricularia oryzae* Cavara in Japan. Jpn. J. Phytopathol..

[30] Xu J.R., Hamer J.E. (1996). MAP kinase and cAMP signaling regulate infection structure formation and pathogenic growth in the rice blast fungus *Magnaporthe grisea*. Genes. Dev..

[31] Zhou S., Liu X., Sun W., Zhang M., Yin Y., Pan S., He D., Shen M., Yang J., Zheng Q. (2021). The COMPASS-like complex modulates fungal development and pathogenesis by regulating H3K4me3-mediated targeted gene expression in *Magnaporthe oryzae*. Mol. Plant Pathol..

[32] Liu H., Lu X., Li M., Lun Z., Yan X., Yin C., Yuan G., Wang X., Liu N., Liu D. (2023). Plant immunity suppression by an exo-β-1,3-glucanase and an elongation factor 1α of the rice blast fungus. Nat. Commun..

[33] Zhang H., Chen Z., Yu Z., Tang L., Gao W., Lu X., Yang J. (2024). *Magnaporthe*-unique gene *MUG1* is important for fungal appressorial penetration, invasive hyphal extension, and virulence in rice blast fungi. J. Fungi.

[34] de Jong J.C., McCormack B.J., Smirnoff N., Talbot N.J. (1997). Glycerol generates turgor in rice blast. Nature.

[35] Cai X., Xiang S., He W., Tang M., Zhang S., Chen D., Zhang X., Liu C., Li G., Xing J. (2022). Deubiquitinase Ubp3 regulates ribophagy and deubiquitinates Smo1 for appressorium-mediated infection by *Magnaporthe oryzae*. Mol. Plant Pathol..

[36] Zhang L., Li D., Lu M., Wu Z., Liu C., Shi Y., Zhang M., Nan Z., Wang W. (2023). Mojmjd6, a nuclear protein, regulates conidial germination and appressorium formation at the early stage of pathogenesis in *Magnaporthe oryzae*. Plant Pathol. J..

