# Supplementary figures and images for: Histone Demethylase MoRph1 Regulates Fungal Development, Pathogenicity, and DNA Damage Repair in Magnaporthe oryzae
Source: J Fungi (Basel). 2026 May 5;12(5):338. doi: 10.3390/jof12050338 (PMC13207933; doi:10.3390/jof12050338)

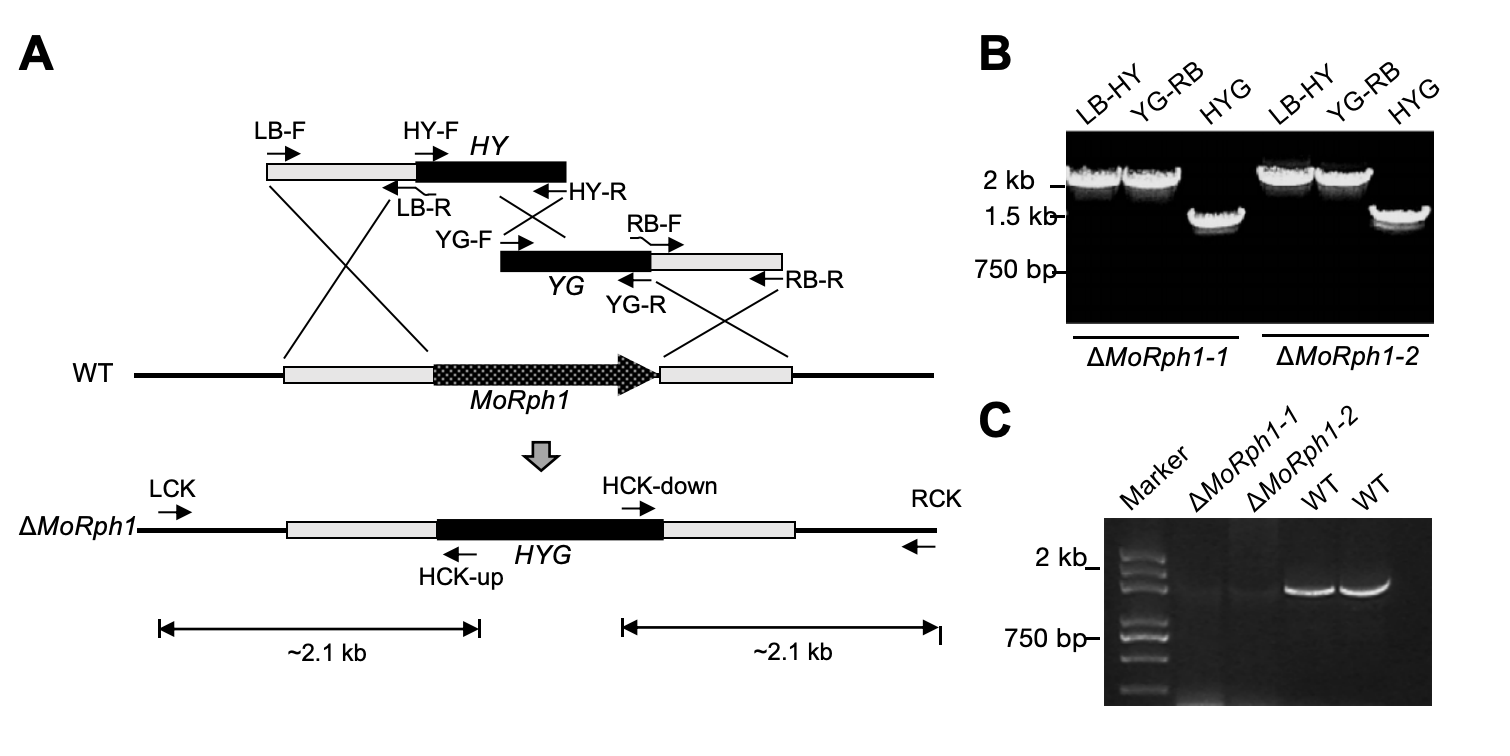

Supplement: Supplementary file 1 [file jof-12-00338-s001.zip › Figure S1.tif]

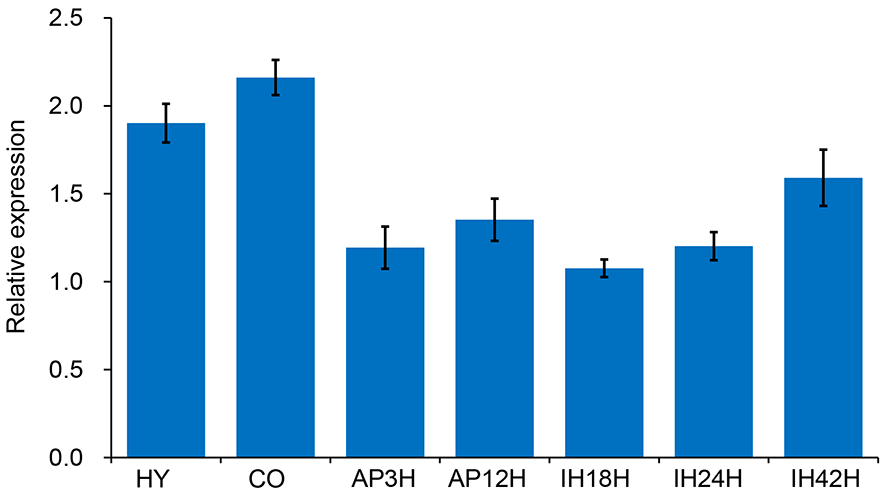

Supplement: Supplementary file 1 [file jof-12-00338-s001.zip › Figure S2.tif]

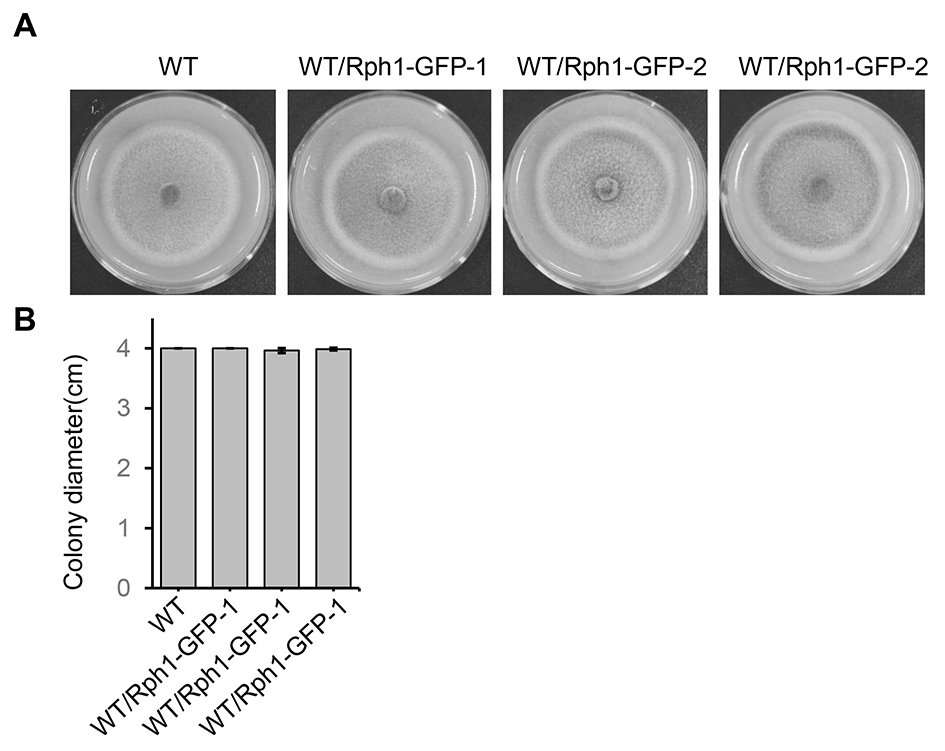

Supplement: Supplementary file 1 [file jof-12-00338-s001.zip › Figure S3.tif]
